# The Role of Oxidative Stress in Pancreatic β Cell Dysfunction in Diabetes

**DOI:** 10.3390/ijms22041509

**Published:** 2021-02-03

**Authors:** Natsuki Eguchi, Nosratola D. Vaziri, Donald C. Dafoe, Hirohito Ichii

**Affiliations:** 1 Department of Surgery, University of California, Irvine, CA 92697, USA; neguchi@hs.uci.edu (N.E.); ddafoe@hs.uci.edu (D.C.D.); 2 Department of Medicine, University of California, Irvine, CA 92697, USA; ndvaziri@hs.uci.edu

**Keywords:** pancreatic β cells, oxidative stress, anti-oxidants, diabetes

## Abstract

Diabetes is a chronic metabolic disorder characterized by inappropriately elevated glucose levels as a result of impaired pancreatic β cell function and insulin resistance. Extensive studies have been conducted to elucidate the mechanism involved in the development of β cell failure and death under diabetic conditions such as hyperglycemia, hyperlipidemia, and inflammation. Of the plethora of proposed mechanisms, endoplasmic reticulum (ER) stress, mitochondrial dysfunction, and oxidative stress have been shown to play a central role in promoting β cell dysfunction. It has become more evident in recent years that these 3 factors are closely interrelated and importantly aggravate each other. Oxidative stress in particular is of great interest to β cell health and survival as it has been shown that β cells exhibit lower antioxidative capacity. Therefore, this review will focus on discussing factors that contribute to the development of oxidative stress in pancreatic β cells and explore the downstream effects of oxidative stress on β cell function and health. Furthermore, antioxidative capacity of β cells to counteract these effects will be discussed along with new approaches focused on preserving β cells under oxidative conditions.

## 1. Introduction

Diabetes is a chronic metabolic disorder affecting 400 million people worldwide and 30.2 million adults aged 18 years or older in just the US [1]. Type 2 diabetes mellitus (T2DM) is characterized by the inability of the pancreatic β cell to produce enough insulin to maintain glycemic control due to increased insulin demand caused by insulin resistance. β cell dysfunction and dedifferentiation, and reduced β cell mass are also suggested to be a central event in the development of the disease [2]. It is well known that hyperglycemia, hyperlipidemia, and inflammation, some of the most common characteristics of a diabetic condition, contribute to β cell damage and dedifferentiation primarily through promoting ER stress, mitochondrial dysfunction, and oxidative stress. Although ER stress and mitochondrial dysfunction have been implicated in β cell dysfunction and apoptosis independently, more recently their pathological role in aggravating β cell oxidative stress has gained particular interest [3]. Pancreatic β cells are particularly susceptible to oxidative stress due to their high endogenous production of reactive oxygen species (ROS) and their low antioxidant capacity, suggesting that oxidative stress may play an important role in β cell failure [4]. Oxidative stress is known to be involved in the pathogenesis of a wide range of diseases ranging from cardiovascular disease to cancer, and extensive studies have been conducted to investigate the effectiveness of potential pharmacological agents targeting oxidative stress [5]. Thus, understanding the molecular mechanisms involved in oxidative stress induced β cell dysfunction may inform novel approaches to treating T2DM.

This review aims to first briefly discuss ways in which toxic environmental factors lead to mitochondrial dysfunction, ER stress, and oxidative stress, and then explore downstream pathways of oxidative stress implicated in β cell dysfunction and death. Lastly, new therapeutic approaches to combat the downstream effect of oxidative stress in β cells will be explored.

## 2. Effect of Environmental Stressors on β Cells

### 2.1. Endoplasmic Reticulum Stress

Due to the increased demand for insulin synthesis and secretion under diabetic conditions, β cells dysfunction is closely associated with ER stress. Proinsulin accounts for 30–50% of cellular protein synthesis of β cells, and approximately 20% of newly synthesized proinsulin fails to reach its native conformation, suggesting a high incidence of proinsulin misfolding [6]. The increased demand overwhelms the ER folding capacity and leads to ER stress. In response, β cell activates the two responses of unfolded protein response (UPR): Adaptive UPR and apoptotic UPR, which work to promote either insulin biosynthesis and secretion or apoptosis. Human islets of patients with T1 and T2DM show increased expression of ATF3 and C/EBP homologous protein (CHOP), pathways of apoptotic UPR, and Bip, an ER chaperone stimulated in adaptive UPR [7]. Supporting this, incubation of human pancreatic β cells under hyperglycemia, hyperlipidemia, and with proinflammatory cytokines results in activation of both adaptive and apoptotic UPR [8,9,10,11,12,13]. In contrast however, more recently, Dai C. et al. demonstrated that the adaptive UPR response was not activated in human β cells under chronic hyperglycemia and hyperlipidemia, suggesting that prolonged glucotoxicity and lipotoxicity may cause defective adaptive UPR response to resolve ER Stress [14]. Several pathways have been implicated in the pathogenesis of ER stress. Both hyperlipidemia and inflammatory cytokines induce ER Ca^2+^ depletion, which impairs chaperones from the protein folding machinery and causes accumulation of misfolded protein [8,10,15,16,17,18]. Additionally, alteration in miRNA profile under proinflammatory treatment and upregulation of cholesterol synthesis under hyperlipidemia are also suggested to contribute to ER stress in human and rodent β cells [19,20,21,22,23].

### 2.2. Mitochondrial Dysfunction

Proper mitochondrial function is crucial to nutrient sensing and insulin secretion in β cells. The increased metabolism as a result of higher glycolytic flux after a meal results in increased cytosolic ATP level. This rise in ATP closes ATP gated K+ channels, which result in depolarization of β cells and the subsequent opening of Ca^2+^ channels, which is coupled to the exocytosis of insulin granules [24]. This signaling cascade is significantly altered in T2DM due to defects in mitochondrial metabolism [25]. Compared to control islets, islets of patients with diabetes mellitus (DM) show reduced glucose stimulated insulin secretion (GSIS), which was associated with lower ATP/ADP ratio, decreased mitochondrial membrane potential, and downregulation of expression of genes associated with energy metabolism [25,26]. Furthermore, mitophagy has also been associated with β cell failure in DM [27]. Mitophagy participates in mitochondrial quality control by selectively removing damaged mitochondria, and malfunction of mitophagy is considered to contribute to β cell destruction. Interestingly, mitochondrial rho GTPase 1 [27], PTEN-induced putative kinase 1, NIP3-like protein X, mitofusion 2, and microtubule-associated protein light chain 3, important mitophagy related genes, have been shown to be augmented in islets of prediabetic patients while significantly downregulated in islets of newly diagnosed and long term diabetic patients, indicating mitophagy impairment in DM [28]. Supporting these observations, transcriptomic and proteomic studies on mice and healthy and diabetic human β cells treated in hyperglycemic and hyperlipidemic conditions demonstrated decreased expression of glycolytic, oxidative phosphorylation, and tricarboxylic acid cycle related genes [9,20,26,29,30,31]. Additionally, both hyperglycemia and hyperlipidemia treatment significantly increased respiration, decreased ATP content, lowered mitochondrial membrane potential, and increased mitochondrial volume, signs that indicate mitochondrial uncoupling and dysfunction [30,32,33,34]. Another study showed both hyperlipidemia and inflammatory cytokine (IL-1β, TNF-α, IFN-γ) treatment altered mitochondria morphology and dynamics through inhibiting mitochondrial fusion, a response to mitochondrial stress important in maintaining health and respiratory efficiency [35,36].

In addition to mitochondrial dysfunction, diabetic condition is closely associated with mitochondria mediated apoptosis. Upon proapoptotic stimuli, cytochrome c is released from mitochondria into the cytoplasm, where it participates in the activation of caspase-9 and subsequent activation of apoptosis executioner caspases 3, 6, and 7 that dismantle the cell. Thus, cytochrome c release from mitochondria is a key step in the initiation of apoptosis. It has been demonstrated that ROS generated by mitochondria trigger apoptosis through a process that involves cytochrome c release in INS-1 cells and mouse β cells [37,38]. Another mechanism by which ROS induces apoptosis is by causing mitochondrial fission. Li F et al. demonstrated that ROS generated by nicotinamide adenine dinucleotide phosphate oxidase (NOX) activation under palmitate treatment of β cell activate ROS-sensitive transient receptor potential melastatin-2 (TRPM2) channels, which subsequently causes abnormal mitochondrial fission [39]. Importantly, silencing of the ROS sensitive TRPM2 channel or treatment of cells with the antioxidant N- acetylcysteine (NAC) in human β cells prevents palmitate induced β cell death and mitochondrial fragmentation, thus highlighting the importance of ROS in stimulating mitochondrial dysfunction and apoptosis [39]. Another mechanism possibly involved in oxidative stress induced apoptosis in human β cells is through the downregulation of Bcl-2, a prosurvival protein [40].

### 2.3. Oxidative Stress

Hyperglycemia, hyperlipidemia, and inflammation are all potent factors contributing to ROS production in β cells. At basal level, ROS in fact plays a crucial role in insulin secretion. It has been demonstrated by Llanos et al. that the ROS generation alongside the moderate Ca^2+^ influx after glucose stimulation is required for RyR channel activation [41]. Once activated, these channels provide the intracellular Ca^2+^ increase required for insulin secretion [41]. Moreover, H_2_O_2_ treatment of islet cells under basal glucose level resulted in augmented insulin secretion, further supporting the concept that ROS plays a critical role in insulin secretion. However, under pathological conditions, the build-up of ROS results in oxidative stress. The major contributor of ROS production is the mitochondrial electron transport chain (ETC). Under hyperglycemic and hyperlipidemic conditions, the increased nicotinamide adenine dinucleotide and flavin adenine dinucleotide levels result in augmented production of ROS through overloading the ETC and causing electrons to leak from complex I and III [42,43,44]. The electrons react with molecular oxygen to form O_2_^•−^, which is quickly converted to H_2_O_2_ [20,26,29,30]. Left undetoxified, the accumulated H_2_O_2_ then could be converted into highly reactive hydroxyl radical and OH^−^ by the Fenton reaction in the presence of higher concentrations of transition metals Cu^2+^ and Fe^2+^ [43]. β cells have several antioxidant defense systems including catalase (CAT), glutathione peroxidase (GPx), thioredoxin (TXN), and periredoxins, which play a significant role in the conversion of H_2_O_2_ into H_2_O and O_2_ [45,46]. However, although these defense mechanisms are present, it has been demonstrated that pancreatic β cells exhibit a lower expression of CAT and GPx1, suggesting higher susceptibility to ROS damage [4]. Several other mitochondrial pathways are considered to be involved in ROS production in β cells under hyperglycemia, which include protein kinase C(PKC) activation, increased intracellular advanced glycation end product formation, hexosamine pathway activation, polyol pathway activation, and oxidative phosphorylation [47,48,49]. Furthermore, hyperlipidemia has also been shown to increase ROS production through activation of NOX, induction of matrix metalloproteinase 2 (MMP2), and stimulation of macrophage infiltration [13,50,51,52,53]. In addition to increasing ROS production, hyperglycemia and hyperlipidemia aggravate oxidative stress through reducing antioxidant capability, evidenced by the reduction in reduced to oxidized glutathione (GSH/GSSG ratio) in hyperglycemia, and reduced superoxide dismutase 1(SOD1) and SOD2 expression in hyperlipidemia in human and rat β cells [14,54,55]. Interestingly, however, in contradiction with the previous studies, in a more recent study by Dai C. et al., hyperglycemic condition did not change superoxide level and antioxidant enzymes levels including SOD1, SOD2, and GPX1 in human islets, while hyperlipidemic conditions showed a significant increase in superoxide and reduction in antioxidant enzyme levels, suggesting that hyperlipidemia may be the major driving force for ROS production [14]. Another study reported similar results showing palmitate treatment significantly induced NO production and lipid peroxidation, major contributors of oxidative stress, while hyperglycemia had no effect [54]. Similarly to hyperlipidemia, inflammatory cytokines increased production of H_2_O_2_ and other ROS products, which modulated activity of NOX and increased expression of genes that recruit macrophage to β cells [4,56,57]. Importantly, it has been proposed that these changes result in chemokine production by β cells, thereby trapping them in an inflammatory environment.

### 2.4. Link between Mitochondrial Dysfunction, ER Stress, and Oxidative Stress

In addition to oxidative stress directly induced by hyperglycemia, hyperlipidemia, and inflammation, mitochondrial dysfunction and ER stress secondary to these conditions can further aggravate ROS production. For example, mitochondrial dysfunction is generally associated with increased ROS production by the organelle itself, which acts to potentiate oxidative stress [58]. One study demonstrated that inflammation results in mitochondrial ROS production, which subsequently activates nuclear factor kappa B (NF-kB) and nitric oxide synthase (iNOS), contributing to oxidative stress through alteration of redox balance and production of NO [59,60]. Mitochondrial ROS production may also further aggravate oxidative stress through contributing to ER stress, evidenced by the reversal of ER stress upon addition of MitoQ, a mitochondrial targeted antioxidant [61]. ER stress is another potent ROS producer and contributes primarily by increasing its oxidative protein folding of proinsulin through its activation of the adaptive UPR, which increases insulin biosynthesis and secretion [62]. Proinsulin folding requires the production of three disulfides for each molecule of proinsulin it synthesizes (3 million disulfides/min per cell), a process which stoichiometrically produces H_2_O_2_ as a ROS by-product [63,64]. Additionally, ER stress can promote ROS generation through contributing to mitochondrial dysfunction. The Ca^2+^ leakage from ER as a result of ER stress causes disruption of Ca^2+^ homeostasis in mitochondria leading to increased mitochondrial ROS production [65].

Interestingly the interaction between the pairs among ER stress and mitochondrial dysfunction, and oxidative stress is not unidirectional. H_2_O_2_ treatment of human islets resulted in the increased mRNA expression of ER stress markers CHOP and P581PK [66]. Furthermore, high oxidative conditions have been shown to play a role in ER Ca^2+^ depletion, which impairs the chaperones of the protein folding machinery and subsequently results in misfolded protein accumulation and ER stress [16]. Oxidative stress also induces mitochondrial dysfunction. In a study using INS-1 cells, H_2_O_2_ treatment resulted in a 4-fold increase in ROS production with a concomitant decrease in ATP production and mitochondrial membrane potential, both of which were reversed by the addition of antioxidant chlorella [67]. Both decreased ATP production and mitochondrial membrane depolarization is associated with impaired insulin secretion [68].

Toxic environmental factors activate several pathways that interact to generate and promote ROS production in β cells. Therefore, the impact of oxidative stress on β cells is a critical factor determining its fate under diabetic conditions.

## 3. Impact of Oxidative Stress on β Cells, Its Downstream Pathways

Oxidative stress has been shown to alter major pathways important for β cell function and survival. Oxidative stress causes AMP-activated protein kinase (AMPK) activation, mammalian target of rapamycin (mTOR) inhibition, and c-Jun N-terminal kinase (JNK) activation in pancreatic β cell. The following section will discuss the impact of these pathways in potentiating β cell dysfunction.

### 3.1. AMPK Activation

**Figure 1:**
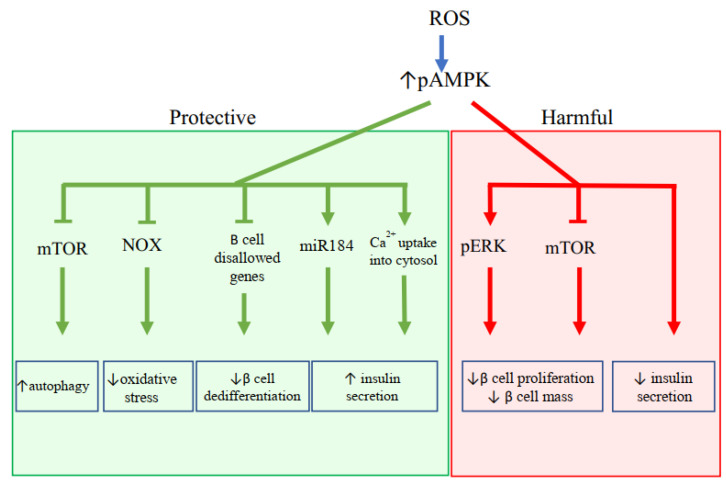
Downstream effects of upregulation of AMP-activated protein kinase (AMPK) activation via oxidative stress. The upregulation of AMPK has both protective and harmful effects. AMPK exerts its protective effect through inhibiting mTOR, NOX2, and β cell disallowed genes, and increasing expression of miR184 and uptake of Ca^2+^. Taken together, they increase autophagy and insulin secretion, and decrease oxidative stress and β cell dedifferentiation. AMPK also has harmful effects mediated by the activation of ERK and inhibition of mTOR, both of which results in decreased β cell proliferation.

AMPK pathway plays essential roles in pancreatic β cells, regulating insulin secretion, metabolic processes, proliferation, and survival. In healthy human and mice β cells, hyperglycemia to glucose stimulation is associated with a clear reduction in phosphorylation of AMPK, indicating reduced activation; however under pathological conditions, this reduction is significantly attenuated [69,70]. This reduced inactivation of AMPK may in part be due to oxidative stress, as oxidative stress has been shown to increase AMPK activation in rodent and human β cell lines [45,71,72]. Short term, this increased activation of AMPK plays both protective and harmful roles in β cells. (Figure 1) First off, Xia G. et al. has demonstrated that ROS mediated AMPK activation in INS-1 cells has been shown to protect β cells through promoting autophagy and reducing oxidative stress [71]. Promotion of autophagy under AMPK activation may be mediated by mTOR inhibition, which is well known to inhibit autophagy, as pAMPK plays a role in mTOR inhibition [73]. Additionally, the reduction in oxidative stress by AMPK activation may be mediated by inhibition of NOX2 which in turn reduces ROS production and JNK1/2 activation [74]. Second, increased AMPK levels may aid in maintaining mature β cell identity, as it has been shown in INS-1 cells that loss of AMPK results in upregulation of disallowed genes, which are often expressed in dedifferentiated β cells or other tissues [75,76]. Third, activated AMPK may also play a protective role by increasing insulin secretion and causing compensatory β cell expansion by the upregulation of miR-184 [77,78]. Furthermore, in another study, pAMPK increased insulin secretion through increased uptake of Ca^2+^ into the cytosol [70]. The increased level of cytosolic Ca^2+^ level under pharmacological AMPK activation may induce insulin exocytosis, and enhance intracellular metabolism, which subsequently increases ATP levels and thus contributes to increasing insulin secretion.

On the other hand, increased pAMPK as a result of oxidative stress may also have harmful effects. Zhang Y et al. showed that ROS mediated upregulation of pAMPK in rat β cells resulted in a downstream increase in extracellular-signal-regulated kinase (p-ERK), which is known to impair β cell proliferation and result in reduced β cell mass [72]. Additionally, pAMPK overexpression increased β cell apoptosis and reduced insulin secretion in mice islet cells [79]. Furthermore, pAMPK may have an inhibitory effect on mTOR, which may be a potent cause of β cell mass loss as mTOR plays a crucial role in maintaining and increasing β cell mass through regulating translation, cell growth, autophagy, proliferation, cell size, and apoptosis [80,81,82]. Lastly, most importantly, although oxidative stress mediated upregulation of pAMPK may contribute to improving β cell function and survival in the short term, long term activation of pAMPK has been shown to have detrimental effects on β cell viability and function. In a study that chronically activated γ2 AMPK, mice β cells showed repressed insulin release, reduced basal β cell activity, and upregulated β cell disallowed gene expression [83]. It is highly probable that oxidative stress although at first may promote β cell survival and function, contributes to pAMPK mediated decline in β cell mass and function as diabetes progresses. Finally, it is important to note that histological studies of islets of patients with T2DM show reduced pAMPK expression [84]. This may suggest that early in DM, the activation of AMPK is upregulated due to several factors including oxidative stress as a protective mechanism, but chronic activation of AMPK results in damage to β cells, and as the disease progresses, eventually to a decline in activated AMPK.

### 3.2. mTOR Inhibition

**Figure 2:**
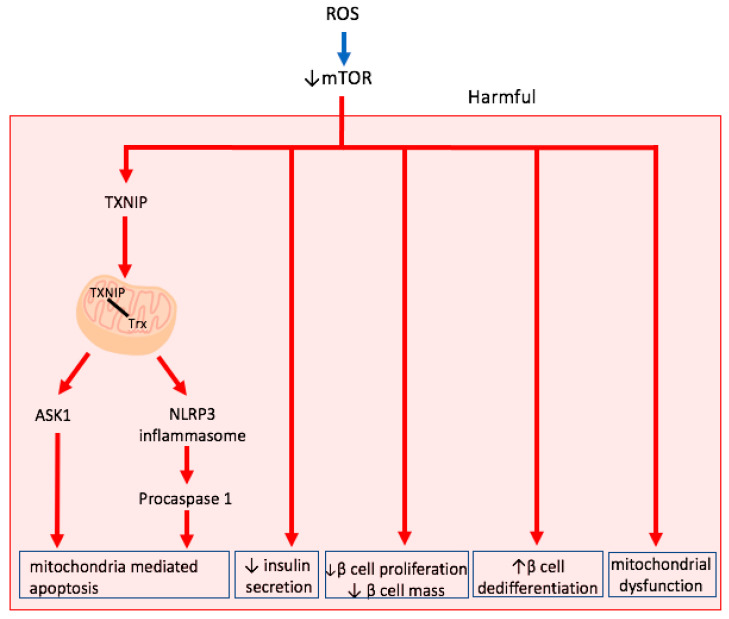
Downstream effects of downregulation of mTOR via oxidative stress. The downregulation of mTOR primarily harms β cells through initiating mitochondrial mediated apoptosis through the upregulation of Thioredoxin-interacting protein (TXNIP), inducing mitochondrial dysfunction, increasing β cell dedifferentiation, and decreasing insulin secretion and β cell proliferation.

mTOR is an evolutionarily conserved, nutrient-responsive serine-threonine kinase that has two functionally and structurally distinct complexes: mTORC1 and mTORC2. Downstream targets of mTORC1 primarily work to stimulate anabolic growth while those of mTORC2 increase proliferation and survival. In general, as discussed earlier, in healthy β cells, mTOR pathway plays an essential role in maintaining β cell mass through regulating cell cycle, autophagy, proliferation, and apoptosis [80]. Although no studies have been conducted in β cell, mTORC1 is ordinarily inhibited under oxidative stress, potentially through the activation of AMPK [82]. The inactivation of mTORC1 may have several detrimental effects (Figure 2), the first of which is the increased expression of Thioredoxin-interacting protein (TXNIP) and shuttling of it into the mitochondria under oxidative stress conditions [85,86,87]. TXNIP is a ubiquitously expressed protein that influences cellular redox balance through negatively regulating the TXN antioxidant systems [88]. In addition to inhibiting reduced TXN, once in the mitochondria, TXNIP can bind to TXN2, thereby releasing apoptosis signal regulating kinase 1(ASK1) from its inhibition and initiating mitochondria mediated β cell apoptosis [86]. Supporting the implication of mTORC1 in this pathway, Maedler K. et al. reported that mouse islet and β cell lines with MTORC1 knockout (KO) showed mitochondrial dysfunction and oxidative stress with an associated increase in TXNIP and carbohydrate-response element-binding protein (ChREBP) [89]. Another mediator implicated in TXNIP induced apoptosis under oxidative stress is NLR family pyrin domain containing 3(NLRP3) inflammasome. It has been demonstrated that TXNIP induced NLRP3 inflammasome assembly leads to the activation of procaspase 1, which then induces cell death through the formation of micropores in the plasma membrane and through interleukin(IL-1β) activation [90,91,92,93]. The importance of the TXNIP/TXN2/ASK1 pathway in mitochondria mediated apoptosis in β cell is supported by the study that showed that TXNIP deficient INS-1 cells are able to prevent mitochondrial death pathway under glucotoxic conditions [94]. Furthermore, TXNIP has an inhibitory effect on TXN, thereby reducing the TXN-dependent enzymatic antioxidant defense against H_2_O_2_ in β cells and potentiating the toxicity of cytosolic ROS [95]. Importantly, ChREBP, as well as TXNIP, have been found to be upregulated in pancreatic autopsy section from patients with T2DM, suggesting that activation of this pathway may be one of the major contributors of β cell pathogenesis in diabetes [85].

Current knowledge of other effects of mTOR inhibition by oxidative stress is very limited. However, studies in mTORKO mice and rapamycin as mTOR inhibitor in β cells allows us to create an idea of the potential effects of mTOR downregulation. For example, it has been demonstrated that β-cell-specific loss of mTORC1 causes DM and β-cell failure due to defects in proliferation, autophagy, apoptosis, and insulin secretion in mice [96]. Furthermore, β cell specific mTOR KO mice as well as mTOR deficient β cell line has revealed compromised mitochondrial membrane potential and respiration, which lead to impaired ATP production, lower intracellular Ca^2+^ levels, impaired insulin secretion, and ROS production [87]. mTOR has also been shown to be important to maintain β cell mature identity and to suppress α cell enriched genes including MAF BZIP transcription factor B (MafB), suggesting a potential role of oxidative stress in β cell dedifferentiation [97]. It is clear that downregulation of mTOR in β cells could potentially have a major detrimental impact on its function and viability. Interestingly, however, mTORC1 has been suggested to be increased under glucotoxic conditions and to cause β cell dysfunction as well [98,99,100]. The effect of hyperlipidemic conditions on mTOR level is less clear. Some studies demonstrate decreased mTOR level with increasing palmitate concentration, while other studies demonstrate increased mTOR level; however, no results reached statistical significance [73,101,102]. Moreover, strangely enough, palmitate treatment partially reversed the mTOR activation in a glucotoxic environment [101]. Thus, combined with the recent study suggesting that only hyperlipidemic conditions resulted in oxidative stress, it is possible that oxidative stress plays a part in the downregulation of mTOR observed under glucolipotoxic treatment; however, more studies directly studying the relation of oxidative stress and mTOR are required to make such argument.

Lastly, it has been shown that human islets of patients with T2D show upregulation of mTORC1 and downregulation of mTORC2 [100]. Thus, it is possible that initial downregulation of mTORC1 in response to oxidative stress may partly play a role in counterbalancing the elevated mTORC1 levels under glucotoxic conditions. Then, later in the disease, possibly through the downregulation of AMPK activation which is evident in patients with DM, mTORC1 is elevated.

### 3.3. Mitogen Activated Protein Kinase(MAPK) Activation (JNK/p38 Activation)

**Figure 3:**
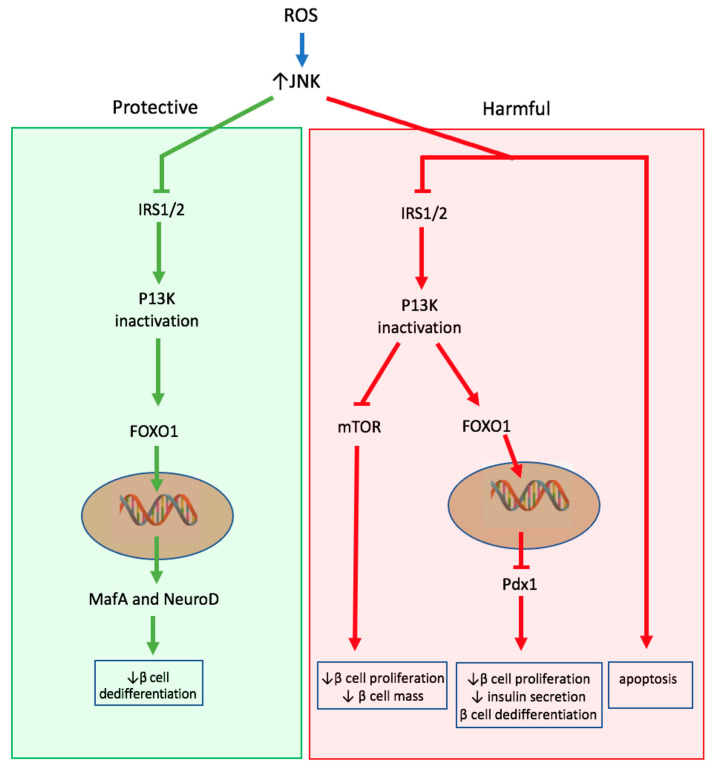
Downstream effects of upregulation of c-Jun N-terminal kinase (JNK) via oxidative stress. The upregulation of JNK have both protective and harmful effects. The upregulation of JNK results in inhibition of the IRS1/2/P13K pathway, resulting in the inhibition of mTOR and nuclear translocation of forkhead box protein O1 (FOXO1). In turn, FOXO1 nuclear translocation results in Pdx1 nuclear exclusion. Overall, these effects result in decreased β cell mass, insulin secretion, and increased β cell dedifferentiation. The upregulation of FOXO1 also has protective effects through its ability to increase expression of β cell mature identity genes including MafA and NeuroD.

Another important pathway activated by oxidative stress in β cells is the JNK pathway. JNK is a MAPK activated under extracellular stress stimuli. In human and rodent β cells, it has been demonstrated by several studies that treatment of β cells with ROS products leads to JNK activation [103,104,105,106]. Furthermore, exposure of human β cells to glucose and leptin activates JNK and induces apoptosis; therefore, the impact of activation of JNK in β cells is of great interest [107]. JNK activation has several downstream effects on β cells that ultimately lead to impaired insulin signaling and apoptosis (Figure 3).

First, JNK impairs insulin signaling through serine phosphorylation and subsequent inactivation of insulin receptor substrate 1/2 (IRS1/2), which results in hindered downstream activation of phosphoinositide 3-kinase (PI3K)/protein kinase B(AKT) pathway in human β cells [105,108,109]. Inactivation of PI3K/AKT pathway has two major detrimental effects on β cells: Reduced activation of mTOR and nuclear translocation of forkhead box protein O1 (FOXO1). As discussed in the previous section, the downregulation of mTOR contributes to loss of β cell mass. Nuclear translocation of FOXO1 exerts harmful effects under oxidative stress through nuclear exclusion of pancreatic duodenal homeobox1 (PDX-1) and by competing for PDX-1 promoter, both of which results in a decreased level of PDX-1 [110,111]. This reduction in PDX-1 stunts β cell proliferation and growth in rodent and human β cells [112]. PDX-1 also plays a crucial role in glucose stimulated insulin secretion by regulating insulin gene expression and associated genes, and preservation of β cell mature identity, suggesting that FOXO1 nuclear translocation contributes to both impaired insulin secretion and dedifferentiation of β cell under oxidative stress [113,114,115]. However, FOXO1 nuclear translocation is not solely harmful to β cells. FOXO1 also plays a protective role under oxidative stress through binding with promyelocytic leukemia protein and Sirtuin 1, leading to upregulation of MafA and NeuroD, important transcription factors that maintain β cell maturity [116].

Secondly, JNK activation may induce human β cell apoptosis. H_2_O_2_ treatment of human pancreatic islets induced β cell apoptosis which was reversed by treatment with exendin-4, glucagon like receptor agonist, through the downregulation of JNK and glycogen synthase kinase-3 beta (GSK3β) activity, suggesting the potential role of JNK in β cell apoptosis [105]. Additionally, another study demonstrated that ROS derived from NOX2 induced β cell apoptosis through JNK, p38MAPK, and p53 pathways [117]. p38 is another MAPK that is activated by oxidative stress and implicated in β cell apoptosis [118]. In another study, however, transgenic mice with JNK overexpression showed that JNK suffices to inhibit insulin signaling in β cells, but is not sufficient to elicit β cell death, suggesting that oxidative stress may induce other factors that concomitantly works with JNK activation to induce apoptosis rather than JNK working alone [119].

It is also important to highlight JunD as a downstream target of JNK that has been shown to be dysregulated in rodent and human β cells under metabolic stress [120,121]. Furthermore, transcriptomics analysis showed that JunD regulates proinflammatory and proapoptotic factors commonly dysregulated in β cell [121]. However, importantly, JNK upregulated JunD only in hyperglycemic and hyperlipidemic conditions, but not under H_2_O_2_ treatment. This shows that the downstream effect of JNK is dependent on the stimuli; further studies directly investigating the downstream effect of JNK activation induced by oxidative stress on β cell are therefore needed to better understand the importance of JNK pathway activation under oxidative stress.

Lastly, it is important to note that although a plethora of studies have demonstrated that NF-κB plays a major role in β cell dysfunction, studies directly investigating the role of oxidative stress in NF-κB activation are limited [122]. One important study by Li X. et al., showed that H_2_O_2_ treatment induced apoptosis in mice β cells, which was reversed by the inhibition of NF-κB inducing kinase (NIK), suggesting the potential role of NF-κB in oxidative stress induced apoptosis [123]. One possible mechanism is through the induction of inflammasome NLRP3 by TXNIP under oxidative stress. NLRP3 deletion in mice β cells showed reduced IL-6, a central downstream effect of IL-1β, suggesting that IL-1β may be a downstream target of NLRP3, which is induced under oxidative stress [92]. The upregulation of IL-1β can in turn activate NF-kB, evidenced by a study that treated human EndoC-βH1 β cells with IL-1β and showed upregulation of NF-kB [4]. This highlights the exhaustiveness of the impact of oxidative stress in β cell dysfunction. Although not directly activating NF-kB, through one of its downstream pathways, oxidative stress is able to activate another major pathway involved in β cell failure.

All in all, oxidative stress can harm β cells through various pathways; therefore, the antioxidative capacity of β cells to counteract these effects is a crucial factor in determining its overall health.

## 4. Antioxidant Properties of β Cells

Gene expression profiling of antioxidative enzymes in different cell types of human pancreatic islets demonstrated a profound deficiency of antioxidant capacity of β cells compared to other cells. First off, superoxide inactivating enzymes SOD1 and SOD2, which were the highest expressed antioxidant enzymes, were 1.4-fold higher in non β cells than in β cells. Even more surprising however, H_2_O_2_ inactivating enzymes GPx and CAT showed 15-fold lower and 3-fold lower expression, respectively, in β cells [4]. Supporting this is another study that investigated the antioxidant capacity of human β cells from patients with T1DM and T2DM, which showed catalase and GPx expression much lower in β cells compared to α cells. Furthermore, they showed that diabetic islet showed significantly lower β/α cell ratio compared to healthy islets and demonstrated that upon exposure to oxidative stress, β cells showed significantly lower survival and viability with increased DNA damage compared to α cells [124]. Interestingly, however, more recently Stancill J. et al. demonstrated that when human EndoC-βH1 cells are exposed to physiologically relevant H_2_O_2_ levels (50 μM) in a continuous manner, β cells are able to detoxify it through peroxiredoxin and thioredoxin antioxidant system [45,95,125]. In contrast, when treated with an H_2_O_2_ bolus (100 μM), β cells are unable to remove it and it results in DNA damage and reduced viability [45]. Furthermore, in comparison to the low expression of GPx and CAT, peroxiredoxin, thioredoxin, and thioredoxin reductase genes are readily expressed in mice and rat β cells [45]. However, a limitation of this study is that it only recorded the effect of physiological H_2_O_2_ levels on β cells for 4 h, thus it will be interesting to see whether prolonged exposure to the physiological H_2_O_2_ level will exhibit similar effects as the bolus or if β cells will be able to continuously neutralize it.

When ROS production exceeds the antioxidant capacity of β cells, Kelch-like ECH Associated protein 1 (KEAP1)/Nuclear factor erythroid 2-related factor 1 (Nrf2)/antioxidant pathway is activated. In a study by Wang J. et al. hyperglycemic rats fed a high fat diet (HFD), and db/db mice showed substantial Nrf2 expression in β cells, while hyperglycemic rats fed HFD and ebselen, an antioxidant, and mice with GPx1 overexpression in β cells prevented Nrf2 expression [126]. Nrf2-keap1 signaling pathway plays a significant role in protecting the cells against various stressors including endogenous and exogenous oxidants. A study investigating the effect of Nrf2 activation by dh404 on human pancreatic islets found upregulation of common antioxidant enzymes including NAD(P)H: Quinone oxidoreductase, Heme oxygenase 1 (HO-1), glucose 6 phosphate dehydrogenase (G6Pd), sulfiredoxin-1, and thioredoxin reductase1 (TXNRD1) [127]. Furthermore, Nrf2 activation decreased the expression of inflammatory mediators and protected human β cells against oxidative stress [127]. Interestingly, a study that evaluated tumor necrosis factor-alpha (TNF-α), Nrf2, and HO-1 levels in normal glucose tolerance, patients with pre-DM, and T2DM found that TNF-α increased Nrf2 and HO-1 decreased as patients became more diabetic, suggesting that, along with aggravation of oxidative stress and inflammatory response, Nrf2 activation and HO-1 expression were both inhibited [128]. One possible explanation of the decline in Nrf2 and HO-1 activity is through the downregulation of PI3K/AKT pathway under conditions of oxidative stress. PI3K/AKT activation is thought to induce Nrf2, and thus its downregulation would continually decrease Nrf2 activation [129].

## 5. New Therapeutic Methods

The complicated nature of downstream pathways of oxidative stress in pancreatic β cells makes it a difficult intervention point. As discussed, oxidative stress increases AMPK activation and mTOR inhibition, and there is a possibility that inhibiting these effects would confer protection against oxidative stress in β cells. However, both decreased AMPK and elevated mTOR activity also display their own associated detrimental effects on β cell function. Thus, we focus on new therapeutic methods regulating mitochondrial function, JNK activation, and antioxidant pathways to improve β cell function. A mitochondria targeted antioxidant MitoQ has been shown to reduce ROS production, O2 consumption, and ER stress markers, and accordingly increase insulin secretion [61]. Anti-diabetic medication such as thiazolidinediones, peroxisome proliferator-activated receptor (PPAR) agonists, have been shown to improve mitochondrial health and increase its biogenesis [130]. In addition to protecting mitochondrial function, PPAR-γ activation with rosiglitazone, antidiabetic drug in thiazolidinediones class, increased insulin secretion, induced FOXO1 nuclear exclusion, and decreased β cell apoptosis in rats [131]. Similarly, incubation of islets of T2D patients with metformin, one of the most commonly used thiazolidinediones, increased β cell insulin content and glucose induced insulin secretion with concomitant decrease in apoptosis and oxidative stress markers [132]. It has also been demonstrated that PPAR-γ activation inhibited cytokine induced JNK activation, which could protect islets from JNK induced dysfunction [133]. Pharmacological JNK inhibitor has also been associated with improved islet survival and function. Ficus Carica leaves extract and cell permeable peptides successfully reduced the expression levels of pJNK with a concomitant reduction in apoptosis-related proteins [134,135]. Lastly, more recently, pharmacological approaches focused on activating Nrf2 pathway have gained momentum. Pharmacological activation of Nrf2 pathway by dimethyl fumarate (DMF), oltipraz, dh404, curcumin, and sulforaphane in human and/or rodent β cells have been shown to protect β cells under different stressors by preserving β cell function and mass [127,136,137,138]. Furthermore, 9 months of curcumin treatment successfully reduced the number of prediabetic patients who advanced to T2DM [139]. Curcumin is considered to impede the progression of diabetes through its antioxidative properties as well as modulation of insulin secretion pathway. An in vitro study that tested the effect of curcumin on human islets reported enhanced expression of common antioxidants including HO-1 and NADPH at both the mRNA and protein level, with a concomitant reduction in β cell apoptosis [140]. Furthermore, Rouse et al. demonstrated that curcumin increased insulin secretion through inhibition of phosodiesterases, thereby increasing the level of cAMP, a crucial component of insulin secretion pathway [141]. In other studies, stimulation of IL-6 protects human β cells from stress induced apoptosis by upregulating autophagy and coupling it with an antioxidant response, an effect mediated by the activation of the Nrf2 pathway [142,143]. Furthermore, more recently, estrogen therapy has been discussed as a potential therapeutic target. Stimulation of estrogen receptors favor islet survival, lipid homeostasis, glucose stimulated insulin biosynthesis and secretion, and proliferation [144]. Silibin preserves β cell mass and function through upregulation of estrogen receptor and subsequent activation of the Nrf2 pathway [145]. Thus, continuing studies on pharmacological Nrf2 pathway activation in β cells hold tremendous preventative and therapeutic potential.

## 6. Conclusions

Oxidative stress plays a critical role in inducing β cell dysfunction and death through the alteration of several important pathways that regulate β cell function and health. Moreover, the ability of oxidative stress to influence apoptotic UPR in ER and mitochondrial apoptosis highlights its destructive role of tying together various pathogenic pathways to further augment the devastative state of β cells. Thus, pharmacological intervention focused on enhancing antioxidative capacity of β cell will play an important role in the preservation of β cells under diabetic conditions.

## Data Availability

Data is contained within the article.

## Abbreviations

T2DM	Type 2 Diabetes Mellitus
ER	Endoplasmic Reticulum
ROS	Reactive Oxygen Species
UPR	Unfolded Protein Response
CHOP	C/EBP homologous *protein*
GSIS	Glucose Stimulated Insulin Secretion
ETC	Electron Transport Chain
CAT	Catalase
GPx	Glutathione peroxidase
PKC	Protein Kinase C
NOX	Nicotinamide adenine dinucleotide phosphate oxidase
MMP2	Matrix metalloproteinase 2
GSH	Reduced Glutathione
GSSG	Oxidized Glutathione
SOD	Superoxide dismutase
UCP2	Mitochondrial uncoupling protein 2
NF-κB	Nuclear factor kappa B
iNOS	Nitric oxide synthase
AMPK	AMP-activated protein kinase
mTOR	Mammalian target of rapamycin
JNK	c-Jun N-terminal kinase
p-ERK	Extracellular-signal-regulated kinase
TXNIP	Thioredoxin-interacting protein
TXN	Thioredoxin
ASK1	Apoptosis signal regulating kinase 1
KO	Knockout
ChREBP	Carbohydrate-response element-binding protein
NLRP3	NLR family pyrin domain containing 3
IL	Interleukin
MafB	MAF BZIP transcription factor B
MAPK	Mitogen activated protein kinase
IRS1/2	Insulin receptor substrate 1/2
PI3K/AKT	Phosphoinositide 3-kinase/Protein kinase B
FOXO1	Forkhead box protein O1
PDX-1	Pancreatic duodenal homeobox1
GSK3β	Glycogen synthase kinase-3 beta
TRPM2	ROS-sensitive transient receptor potential melastatin-2
NAC	N-acetylcysteine
NIK	NF-κB inducing kinase
KEAP1	Kelch-like ECH Associated protein 1
Nrf2	Nuclear factor erythroid 2-related factor 1
HFD	High fat diet
HO-1	Heme oxygenase 1
G6PD	Glucose 6 phosphate dehydrogenase
TXNRD1	Thioredoxin reductase1
TNF-α	Tumor necrosis factor-alpha
PPAR	Peroxisome proliferator-activated receptor
DMF	Dimethyl fumarate
